# The Chain of Cannulation: A Qualitative Comparison of the Cannulation Process and European Guidelines

**DOI:** 10.1111/jorc.70035

**Published:** 2025-10-31

**Authors:** Karin Staaf, Fredrik Uhlin

**Affiliations:** ^1^ Department of Health, Medicine and Caring Sciences Linköping University Linköping Sweden; ^2^ Department of Nephrology Linköping University Hospital Linköping Sweden; ^3^ Department of Health Technologies Tallinn University of Technology Tallinn Estonia

**Keywords:** cannulation technique, complications, haemodialysis, hygiene, nursing

## Abstract

**Background:**

The cannulation of an arteriovenous fistula can be performed using several different techniques and surrounding routines, which are presently not well‐defined. The recently described chain of cannulation includes planning cannulation, pre‐cannulation, during cannulation, evaluating cannulation and post‐cannulation, and was based on the Swedish context, in which the buttonhole cannulation technique is the most common.

**Objectives:**

To explore whether the chain of cannulation is in agreement with nursing guidelines from other dialysis contexts, in terms of cannulation techniques and their preconditions.

**Design:**

Qualitative comparison of guidelines regarding arteriovenous fistula cannulation.

**Participants:**

Two guidelines regarding the process of arteriovenous cannulation, written in English, were included and compared to a compilation of Swedish local guidelines.

**Measurements/Approach:**

The British and European guidelines were analysed using deductive qualitative content analysis. The utilised categorisation matrix was based on the chain of cannulation and its subcategories. The identified subcategories were compared among guidelines, using qualitative triangulation to find agreement, dissonance or silence.

**Results:**

The majority of subcategories showed partial agreement between at least two of the guidelines. Silence and dissonance were identified in the following subcategories: hygiene routines, preventing pain, how to needle, angle during cannulation, needle withdrawal and haemostasis. Two new subcategories were identified in the British guidelines: patient involvement, and screening and control.

**Conclusion:**

The chain of cannulation, which originated in the Swedish context, showed agreement with needling guidelines from other nursing contexts regarding arteriovenous cannulation techniques and their preconditions.

## 1 Introduction

For patients who require haemodialysis, the arteriovenous fistula (AVF) is the most common and beneficial access to the blood stream (Lok et al. 2020; Schmidli et al. 2018). Thus, it is important to take care of the AVF to prevent complications and prolong its patency. An essential component of caring for the AVF is performing cannulation in an appropriate manner. Patients typically come to a dialysis unit for treatment three times per week. For each treatment, the AVF is cannulated with two needles—one to draw the blood from the patient to the dialysis machine (arterial) and one to return the blood to the patient (venous)—adding up to more than 300 cannulations in a year.

## 2 Literature Review

There is no established definition of the AVF cannulation procedure and the suboperations it includes. Among studies examining cannulation technique, the majority have compared two or three of the most common methods of inserting the needle in the vessel. The two most frequently compared cannulation techniques are the buttonhole (BH) technique, where a blunt needle is inserted into exactly the same site at each treatment, and the rope ladder (RL) technique, where cannulations are systematically performed along the whole vessel (L. Harwood et al. 2017). BH has also been compared to the so‐called standard technique, which is a mix between RL and the area puncture (AP) technique (C. A. Fielding et al. 2022), where the needle is cannulated at a new site for each treatment, but all new sites are within the same limited area (Parisotto and Pancirova 2018). Some studies have also described different components of the cannulation process and the dialysis treatment, such as haemostasis after needle withdrawal (Sallée et al. 2020), ultrasound use during cannulation (Schoch et al. 2020) and orientation of the bevel during cannulation (Ozen et al. 2022).

Although there is no accepted standard for which routines to include in AVF cannulation or how to define cannulation techniques, some studies have tried to define successful cannulation. Van Loon et al. (2010) describe successful cannulation as that performed using two needles, without miscannulation and which enables dialysis with ordinary blood flow. Marticorena and Donnelly (2016) add that the needle should penetrate through the skin and into the vessel in one single stroke. L. E. Harwood et al. (2016) and Wilson and Harwood (2017) asked patients and dialysis providers to describe a successful needling. They found that patients evaluated success in terms of their emotional response to pain and anxiety, a ‘friendly’ nurse–patient relationship, a technically skilled nurse and the impact of the environment. To achieve success in these terms, dialysis providers described the need for patient‐centred care, teamwork, and nurses' skill and self‐awareness.

Successful cannulation can refer to only one single cannulation or a certain number of cannulations (Marticorena and Donnelly 2016). To widen the term, there are also descriptions of good cannulation technique (M. Aitken et al. 2018). Good techniques are those that are quick and easy to perform (Wilson and Harwood 2017), while still preventing AVF complications and preserving AVF function (Viecelli et al. 2020).

It has been established that cannulation includes more than only the needle insertion. Professor Bernad Canaud described cannulation in the AVF as an art and reliant on the craftsmanship of nurses (Parisotto and Pancirova 2018). Van Loon et al. (2010) described suboperations—for example, the needle type and use of tourniquet—as characteristics of cannulation practice, and used them to predict time to unsuccessful cannulation. McCann et al. (2009) described different elements of cannulation for the purpose of education. In a systematic review, C. A. Fielding et al. (2022) compared the different cannulation techniques and concluded that the outcomes of various techniques could not be separated because the suboperations were not described in detail. To enable comparison between the different methods of needle insertion, there must be a standard for reporting components of the process.

**Figure 1:**
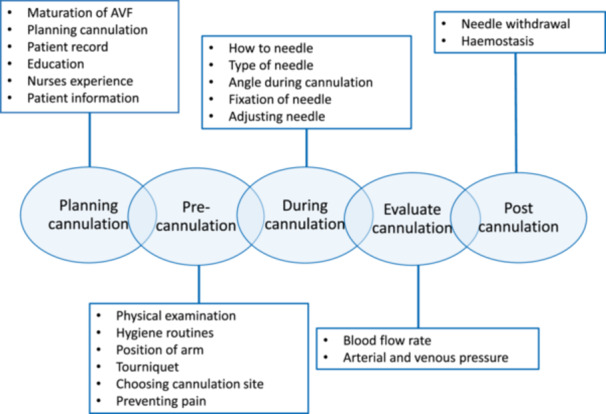
The chain of cannulation as described by Staaf et al. (2023b).

To date, the different parts of the AVF cannulation process are not described as well as the actual cannulation. Staaf et al. (2023b) described the process and its preconditions as the chain of cannulation (Figure 1). Their study was performed from a Swedish perspective, and in Sweden, the majority of cannulations in the AVF are performed using BH. It is unclear whether the cannulation process and its preconditions are described in a way that is comparable to those in countries where RL and AP are the preferred techniques. To be able to apply the chain of cannulation in studies where needle insertion routines other than BH are used, or in dialysis units where RL or AP is preferred, this process must be compared with guidelines from other countries. In the present study, we aimed to explore whether the chain of cannulation corresponds with nursing guidelines from other dialysis contexts, in terms of AVF cannulation technique and its preconditions.

## 3 Materials and Methods

### 3.1 Design

This study is a qualitative comparison of three different guidelines regarding AVF cannulation.

### 3.2 Data Collection

We used the descriptions of the chain of cannulation published by Staaf et al. (2023b), as well as two other guidelines—one British (M. Aitken et al. 2018) and one European (Parisotto and Pancirova 2018). All three were found using convenience sampling. They were well known in our clinic and used during education in AVF care. The latter two guidelines were written in English and address dialysis providers that predominantly use the AP and RL techniques (Parisotto et al. 2014). All included guidelines cover the whole cannulation process, as well as the different cannulation techniques and how to perform them.

### 3.3 Analysis

We performed deductive qualitative content analysis (Elo and Kyngäs 2008). This method was chosen as it can be used to analyse large amounts of text as well as compare this text with earlier knowledge. A categorisation matrix was created, which included the categories and subcategories from Staaf et al. (2023b). All three guidelines were compared and coded to this matrix, one by one. Content that did not fit into a predetermined category was placed in the category ‘other’, which was then inductively analysed, yielding the emergence of two new subcategories (Table 1).

**Table 1:** Overview of the deductive qualitative content analysis according to Elo and Kyngäs (2008).

Phase	Steps of analysis process	Describing how
Preparation	1. Choice of the unit to analyse	One to three (or more) sentences describing the same content.
	2. Manifest analysis only	Only the content in the written words was analysed.
	3. Become acquainted with data	Read the text several times to learn to know the text.
Organisation	4. Creating analysis matrix	Categories and subcategories from ‘chain of cannulation’ were used.
	5. Data coding according to categories in analysis matrix	Units from the text were coded with the categories/subcategories from ‘chain of cannulation’.
	6. Data that did not fit in the analysis matrix was analysed using inductive analysis; open coding of units, grouping of codes and categorisation	The text marked with the code ‘Other’ was parted in units due to its content and marked with codes. Codes were grouped, and the two categories emerged: patient involvement and screening and control.
	7. Abstraction of new categories and subcategories from Step 6 and comparison of results from Step 5.	Text sections were condensated into meaning units.
Reporting	Description	Condensed text from the three guidelines was compared.

The parts of the text that were coded to a subcategory (text section) were condensed into meaning units (Table 2). Then the meaning units from each of the three guidelines were compared based on qualitative triangulation, according to Farmer et al. (2006), to examine whether there was agreement, dissonance or silence among separate subcategories.

**Table 2:** Examples of a category, subcategories, text sections and meaning units as a part of the analysis.

Category	Subcategory	Text section	Meaning units
Planning cannulation	Education	(H.1 7) If the patient is planning to insert their own needles, they should be encouraged to develop the track themselves. When patients insert the needles themselves, they will approach the vessel at a different angle than when another person performs this. By developing their own tracks, patients will ensure greater success when inserting blunt/dull needles themselves and will develop the skills required to troubleshoot problems in later needle insertions. [BH]	Teach the patient to self‐cannulate while creating the BH track. Then they can adapt the angle according to their own choice and learn how to create tracks.
	Planning cannulation	(F.3 2) The needling plan should include paper documentation that must be completed by all haemodialysis nursing staff who are inserting the needles. It should be designed to promote communication to ensure consistent needle insertions between staff members. This record is less necessary and not mandatory when the same person inserts the needle each time. However, members of a haemodialysis nursing staff will always rotate between shifts, and sometimes be unavailable, including unplanned leave.	The plan is a paper document, which all needling staff should read from, and write on and develop. The paper should be a means of communicating between staff. Therefore, this paper is less necessary if cannulation is performed by one single person (who is not a part of a staff team).

Abbreviation: BH, buttonhole.

### 3.4 Ethical Considerations

This research does not include human participants; therefore, ethical approval is not required.

## 4 Results

Of the three analysed guidelines, two described all subcategories in the chain of cannulation. Although the main content was the same among the guidelines, the descriptions often included slightly different content; therefore, the overall assessment was that they showed ‘partial agreement’. In the British guidelines (M. Aitken et al. 2018), we identified two new subcategories that were not found in the other two guidelines (Table 3).

**Table 3:** The chain of cannulation, and whether the different guidelines exhibited agreement.

Category	Subcategory	Agreement, dissonance or silence
Planning cannulation	Maturation and cannulation in new AVF	Partial agreement
	Patient record	Partial agreement
	Education and experience	Partial agreement
	Patient information	Partial agreement
Pre‐cannulation	Physical examination	Partial agreement
	Hygiene routines	Partial agreement and dissonance
	Arm position	Partial agreement
	Tourniquet	Partial agreement
	Choosing a cannulation site	Partial agreement
	Preventing pain	Silence and dissonance
During cannulation	How to needle	Partial agreement and dissonance
	Type of needle	Partial agreement
	Angle during cannulation	Partial agreement and dissonance
	Fixating and adjusting	Partial agreement
Evaluating cannulation	Arterial and venous pressure	Partial agreement
	Blood pump speed	Partial agreement
Post‐cannulation	Needle withdrawal	Silence and partial agreement
	Haemostasis	Silence and partial agreement
Other	Patient involvement	Silence
	Screening and control	Silence

*Note:* The first five categories are a part of the chain of cannulation. The category ‘other’ includes areas that were new to the categorisation matrix.

### 4.1 Planning Cannulation

The three guidelines exhibited partial agreement in all four subcategories of planning cannulation. For example, they agreed that an experienced nurse should perform the first cannulation and that one or two needles can be placed in the AVF during the first cannulation. However, the three guidelines also somewhat differed in content and what they emphasise. Both Parisotto and Pancirova (2018) and Staaf et al. (2023b) mention that when using a new AVF, in a patient who already has a central venous catheter (CVC), the CVC can be used as a substitute for the artery or venous needle. M. Aitken et al. (2018) do not describe this method. On the other hand, only M. Aitken et al. (2018) mention that RL is the first technique that should be used with all new AVFs; AP should be avoided, and BH should not be introduced until the AVF is fully matured (> 6 mm vein diameter at both sites).

The three guidelines also agreed that the patient's record should be used as a tool for communication and information sharing. M. Aitken et al. (2018) and Parisotto and Pancirova (2018) both described the design of a needling plan, and all three guidelines explained what AVF‐related content should be included in the patient's record.

All three guidelines agreed that regular training in needling is important. M. Aitken et al. (2018) and Parisotto and Pancirova (2018) also highlighted the need for regular assessment of cannulation skill. Moreover, M. Aitken et al. (2018) emphasised the importance of discussing the different cannulation techniques, and their pros and cons, during education, which they think could lead to a reduction of AP in favour of RL. Good needling technique is based on experience, and this experience should be shared with colleagues that lack experience regarding all three cannulation techniques. M. Aitken et al. (2018) also noted that only experienced senior registered nurses should use POCUS, due to the difficulty of interpreting the images.

All three guidelines agreed that patients should be provided with information about cannulation techniques and AVF care, both orally and in writing. Staaf et al. (2023b) and Parisotto and Pancirova (2018) highlighted the importance of teaching the patient about hygiene and how to stop acute bleeding. They also described the possibility of patients wearing a bracelet to let others know they have an AVF. On the other hand, M. Aitken et al. (2018) highlighted the importance of patients having information about the different cannulation techniques and ways to handle needle anxiety.

### 4.2 Pre‐Cannulation

The three guidelines showed partial agreement on all pre‐cannulation subcategories, except two: hygiene routines and preventing pain. Regarding hygiene, we found both partial agreement and dissonance. All three guidelines agreed that personal protective equipment (PPE) should be used and that the AVF arm should be washed with soap and water before treatment. If BH is implemented, the scab should be removed, and disinfection should be performed before and after removal, and after any time the disinfected area is touched.

Dissonance was found on other topics. For example, when BH is used, M. Aitken et al. (2018) recommended not moistening the scab before removing it, while Parisotto and Pancirova (2018) recommended moistening the scab to remove it whole. Additionally, M. Aitken et al. (2018) recommended the use of mupirocin ointment in patients undergoing BH and having a high infection risk, while this technique was not mentioned by Parisotto and Pancirova (2018) or Staaf et al. (2023b). Staaf et al. (2023b) noted that chlorhexidine 5 mg/mL is recommended in Sweden. On the other hand, M. Aitken et al. (2018) recommended chlorhexidine 20 mg/mL but were uncertain about the proper strength. Povidone iodine and octenidine dihydrochloride were also mentioned. Parisotto and Pancirova (2018) referred to local guidelines for further information regarding the issue of what disinfectant to use. We also identified differences regarding how to apply the disinfectant. Parisotto and Pancirova (2018) recommended disinfecting in circular motions. Staaf et al. (2023b) reported that Swedish units disinfect both with a circular motion and by rubbing back and forth.

Needle‐related pain is described by Staaf et al. (2023b) and M. Aitken et al. (2018), while Parisotto and Pancirova (2018) do not mention pain prevention. Staaf et al. (2023b) and M. Aitken et al. (2018) illuminate the problem of needle‐related pain from different perspectives. Staaf et al. (2023b) noted that nurses should inform the patient about the possibility of analgesic and allow patients to apply it themselves if they want to. On the other hand, M. Aitken et al. (2018) described several alternative approaches for handling pain and needle anxiety, including the use of a tourniquet and BH. They also highlighted the importance of asking about the patients' perspectives regarding pain.

All three guidelines recommended physical examination of the AVF ahead of dialysis. M. Aitken et al. (2018) and Parisotto and Pancirova (2018) agreed that this assessment should be performed by ‘look, listen, feel’. Staaf et al. (2023b) reported that some units recommend this method, while others recommend only to look and feel. Additionally, Staaf et al. (2023b) and Parisotto and Pancirova (2018) recommended regular blood flow measurements (e.g., via ultrasound dilution technique). M. Aitken et al. (2018) instead proposed an in‐depth assessment every one to third month (e.g., clinical clues like achieved blood flow rate and pressure parameters, physical examination, signs or symptoms and periodic surveillance like recirculation and blood flow rate via ultrasound dilution technique).

M. Aitken et al. (2018) noted certain signs that should be paid attention to when BH is used—including hubbing, signs of infection, enlarged needling sites, prolonged bleeding or pain/discomfort and skin integrity. Staaf et al. (2023b) and M. Aitken et al. (2018) recommended that the same arm position should be used for every cannulation with BH. Notably, letting the patient remember this arm position can be a way to let the patient participate in their care (M. Aitken et al. 2018). All guidelines described the use of a tourniquet to varying extents. Staaf et al. (2023b) indicated that tourniquet use is mandatory in some units and voluntary in others. M. Aitken et al. (2018) recommended tourniquet use for all patients to enlarge the vessel and prevent pain. Parisotto and Pancirova (2018) indicated that AVF longevity is improved if pressure is applied by the patient's own fingers instead of a medical tourniquet.

All three guidelines agreed that needles should be placed 3–8 cm from each other. When RL is used, the distance from the last cannulation site should be 3–10 mm. M. Aitken et al. (2018) additionally mentioned that the full vessel length should be used for RL, and Parisotto and Pancirova (2018) highlighted the importance of not using the same site within 2 weeks.

### 4.3 During Cannulation

The greatest dissonance was observed regarding recommendations for the technique used while inserting the needle. Parisotto and Pancirova (2018) recommended RL as the first choice. M. Aitken et al. (2018) recommended using either RL or BH based on local expertise, condition of the AVF and the patient's preference. Both of these guidelines also described how these different techniques are performed. Staaf et al. (2023b) reported that the majority of units and nurses recommend BH to most patients. Swedish local guidelines usually only describe BH and do not describe the other techniques (although some do). We also identified dissonance regarding sharp needles when BH is performed. Staaf et al. described dialysis units where sharp needles were used all the time, and others in which sharp needles are only used for creation of a new track. M. Aitken et al. (2018) indicated that sharp needles are only allowed for new track creation in BH and that anyone who needs to use a sharp needle should cannulate at least 2 cm from the track.

Regarding needle size, Staaf et al. (2023b) reported recommendations of 17–16 G in new AVFs and 15 G for mature ones. Parisotto and Pancirova (2018) recommend that the needle gauge be adapted to the desired blood flow. M. Aitken et al. (2018) added that adaption should also be made to the size of the vessel. Both Staaf et al. (2023b) and M. Aitken et al. (2018) mentioned plastic cannulas. Staaf et al. (2023b) reported that some units recommend plastic cannulas for new AVFs, while M. Aitken et al. (2018) mentioned the use of plastic cannulas when limb movement should be allowed and sometimes during BH track creation.

Both Parisotto and Pancirova (2018) and Staaf et al. (2023b) recommended an angle of 20–35 degrees during cannulation, while M. Aitken et al. (2018) recommended always adapting the angle according to the depth. Parisotto and Pancirova (2018) indicated that the arterial needle can be directed either anterograde or retrograde, while the venous needle should always be cannulated anterograde. Anterograde position is preferred to prolong AVF survival. M. Aitken et al. (2018) recommended anterograde cannulation with both the arterial and venous needle, but stated that retrograde cannulation can be performed at the arterial site when necessary. According to Parisotto and Pancirova (2018), the bevel of the arterial needle should face down if the direction is anterograde, but up if the needle is placed retrograde. Staaf et al. (2023b) reported that some units recommend the bevel face up and others down. M. Aitken et al. (2018) only recommended that the bevel face up.

All three guidelines describe techniques for needle fixation. M. Aitken et al. (2018) and Parisotto and Pancirova (2018) called these techniques butterfly/chevron and H. Both M. Aitken et al. (2018) and Staaf et al. (2023b) indicated that the cannulation site could be covered by tape, but that it is not necessary. Staaf et al. (2023b) added that moist tape should be changed, and that tubing should be fixed by either clothing, bedding or hand to prevent venous needle dislodgement.

### 4.4 Evaluating Cannulation

All three guidelines described the importance of monitoring of arterial and venous pressure. This should be combined with monitoring of blood flow rate (Parisotto and Pancirova 2018; M. Aitken et al. 2018; Staaf et al. 2023b). Parisotto and Pancirova (2018) and Staaf et al. (2023b) reported that normal blood flow rates are 300–350 or 250–350 mL/min, respectively. M. Aitken et al. (2018) described that blood flow rate will differ according to needle size, and that needle size depends on the AVF width—with thinner needles and thinner veins leading to lower blood flow.

### 4.5 Post‐Cannulation

Only two of the guidelines included recommendations regarding needle withdrawal and haemostasis (Parisotto and Pancirova 2018; Staaf et al. 2023b). Both agreed that compression should be performed using sterile gauze for 5–15 min. However, Parisotto and Pancirova (2018) recommended that pressure should be applied using two fingers to cover both the hole in the skin and the hole in the vessel. Staaf et al. (2023b) reported that some local guidelines agreed with this recommendation, but the majority described compression using only one finger per site.

### 4.6 Other

We found two new subcategories in the guidelines of M. Aitken et al. (2018) compared to those in the categorisation matrix. These subcategories were patient involvement, and screening and control. The British guidelines encouraged dialysis providers to involve the patient and described how patients can participate in cannulation and caring for the AVF. This is not described in the guidelines of Parisotto and Pancirova (2018) and Staaf et al. (2023b), which only described the importance of patient information (planning cannulation). Additionally, the recommendations of M. Aitken et al. (2018) were the only ones to describe the importance of MRSA/MSSA screening to achieve infection control and counter antibiotic resistance.

## 5 Discussion

The present analysis revealed substantial agreement between the chain of cannulation and needling guidelines regarding AVF cannulation techniques and their preconditions. However, although the content was largely similar between the three analysed guidelines, the different preconditions were often described somewhat differently (partly agreement). Other cannulation‐related topics were handled in opposite manners between recommendations (dissonance) or were not mentioned at all in some guidelines (silence).

Cannulation of AVFs is an important part of nursing care during haemodialysis treatment. The selection of a technique and its performance are mainly based on clinical experience. Research in this area has only been performed during the last 20 years. Cannulation technique has predominantly been studied through comparisons between two methods of inserting the needles into the vessel (L. Harwood et al. 2017). Therefore, positive and negative aspects of cannulation often are associated with the different cannulation techniques—for example, RL is believed to cause more infiltrations, while BH leads to more infections. To prevent a certain kind of complication, one can select the technique with a lower risk of this complication. However, cannulating nurses are aware that the needling technique includes steps beyond only the needle insertion, as has been described in earlier studies (Parisotto et al. 2014; C. A. Fielding et al. 2022). Guidelines—like those analysed in the present study—also convey this broader perspective of cannulation. To distinguish between the two, we therefore suggest that the needle insertion be referred to as a cannulation technique while the broader perspective ought to be described as a cannulation process or cannulation. Regardless of the selected needle insertion technique, infiltrations and infections can be decreased by a slight change in the cannulation process—for example, by using POCUS during more difficult cannulation (Eves et al. 2021) or applying different hygiene routines (O'Brien et al. 2012; Labriola et al. 2024). Thus, it is important to consider all of the steps during cannulation when investigating different cannulation techniques.

Our present comparisons mostly revealed similarities or partial agreement among the different guidelines. However, the differences that we identified may be of greater importance. One of the greatest disparities was in how the different cannulation techniques were described. The guidelines of both Parisotto and Pancirova (2018) and M. Aitken et al. (2018) described the different techniques and recommended when and how to use them. On the other hand, Staaf et al. (2023b) reported that the local guidelines were mainly focused on BH.

**Figure 2:**
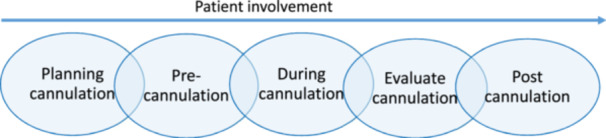
The adapted chain of cannulation.

All three guidelines describe most of the recommendations from a general perspective, which is important to take into account regardless of the cannulation technique chosen. Other recommendations differ between the cannulation techniques described (Supporting Information S1). Comparing the clinical recommendations provides an opportunity to develop the cannulation routines on the local dialysis unit by incorporating more evidence and descriptions of how the steps should be performed. By adding the two new subcategories, found in M. Aitken et al. (2018), into the chain of cannulation, the cannulation process can be studied from this perspective. Only M. Aitken et al. (2018) explicitly described the importance of patient involvement and how to increase patient participation in cannulation. Nursing care can be described as one part action, and one part relationship (Griffin 1980). The subcategories in the chain of cannulation are of the action kind. The required actions may be performed by a dialysis provider or by a trained patient on home haemodialysis. However, according to L. E. Harwood et al. (2016) and Wilson and Harwood (2017), successful needling should also involve patient‐centred care, the emotional response to pain and anxiety, and a friendly nurse–patient relationship. Thus, the nurse–patient relationship should also be a part of the chain of cannulation. M. Aitken et al. (2018) described the addition of patient involvement, expanding on the action and relationship components of the nursing care. Studies show that patients want to participate in their care; however, staff and patients often have different views of participation (Årestedt et al. 2019) and the optimal degree of patient involvement (Martinsson et al. 2021). Although patient involvement was not mentioned in the local guidelines described by Staaf et al. (2023b), it was illuminated in the mixed‐method study based on the same survey (Staaf et al. 2023a). Dialysis nurses expressed that they selected a cannulation technique according to their own and their colleagues' experiences, local guidelines and the patient's preference. The chain of cannulation should therefore be expanded to also include patient involvement (Figure 2). Screening and control become a subcategory to planning cannulation.

Pain management during cannulation was described in different ways in the guidelines of Staaf et al. (2023b) and M. Aitken et al. (2018), but not at all in those of Parisotto and Pancirova (2018). Pain during cannulation is commonly described in qualitative studies about patients' experiences of needling and dialysis care (Casey et al. 2014; C. Fielding et al. 2023). Pain during cannulation is reported in 12%–80% of cannulations and is strongly associated with fear and a decreased quality of life (Kosmadakis et al. 2021). Therefore, it may be appropriate for cannulation technique guidelines to highlight pain and pain relief. Needle‐related anxiety may also be an important component of the sensation of pain (Duncanson et al. 2024) and should also be highlighted in guidelines to make dialysis providers aware of its common occurrence and possible ways to prevent it.

Pain relief during cannulation has been described previously in the literature (E. Aitken et al. 2013; Alzaatreh and Abdalrahim 2020; Zhang et al. 2020). Although the recommendations for pain relief are described differently in M. Aitken et al. (2018) and Staaf et al. (2023b), they are not contradictory. Instead, they could be combined to relieve pain from different perspectives and give the patient a more positive cannulation experience.

Recommendations on whether the needle bevel should be cannulated up or down differ in the compared guidelines. Studies suggest that cannulation downwards may result in less pain, shorter haemostasis after needle withdrawal and fewer injuries to the posterior vessel wall (Li et al. 2014; Ozen et al. 2022; Yilmaz et al. 2022). However, these studies have been conducted on AP and RL. It is therefore unclear which direction the bevel should be facing when using the BH.

The present analysis has several limitations. First, as this study involves the comparison of guidelines, partly based on expert opinions, it cannot itself be used as a description of how to perform different cannulation routines, only as a tool that can outline the elements that need to be described during the cannulation process. Second, the three guidelines compared in this study all come from Europe. The results may have been different if the study had included non‐English guidelines. However, our results confirm the consistency of the chain of cannulation and its parts, despite slight differences in the routines and cannulation techniques among different dialysis units and nations. It is likely that units create their own routines based on traditions and proven experience, due to the lack of published evidence. There remains a need for further research on different preconditions and different parts of the chain of cannulation to increase the quality of AVF care and improve patients' quality of life and AVF patency.

### 5.1 Implications for Clinical Practice

As the chain of cannulation describes the different preconditions of cannulation techniques, and concretises the cannulation process, it may serve as a template when new guidelines are written and what subjects ought to be included to describe all perspectives of this process, both as a whole and in the different parts of the chain. By using the same structure, guidelines describing the same process may be easier to compare, discuss and adapt to one's own context. The chain of cannulation may, for example, be used as a tool for discussion among dialysis providers on the same unit or as a starting point in a workshop. This allows the dialysis providers to reflect, more in detail, upon what aspects of cannulation technique are important to develop and which steps are already working properly and reliably. It may also be a useful component of training for new dialysis providers and self‐cannulating patients, as it describes a structure of all the routines included in the cannulation process. More experienced dialysis providers may use the compilation to compare how cannulation is performed in different dialysis units and countries and develop their cannulation process more based on evidence. The chain, as a whole or in parts, can also inspire further clinical studies to gather evidence regarding the routines that are currently built only from traditions and proven experience.

## 6 Conclusion

The five categories in the chain of cannulation are applicable regardless of the cannulation technique used. The subcategories can serve as a tool or a checklist when developing new guidelines or planning research related to cannulation technique. The chain of cannulation can also be used to illuminate the similarities and differences between various cannulation techniques, making them valuable for educational purposes and further training.

## Author Contributions

Research idea and study design: K.S. Data acquisition: K.S. Data analysis/interpretation: K.S. and F.U. Supervision/mentorship: F.U. Each author contributed important intellectual content during manuscript drafting or revision.

## Ethics Statement

This research does not include human participants; therefore, ethical approval is not required.

## Conflicts of Interest

The authors declare no conflicts of interest.

## Supporting information

Supporting Information.
