# Supplementary material for: The Chain of Cannulation: A Qualitative Comparison of the Cannulation Process and European Guidelines
Source: J Ren Care. 2025 Oct 31;51(4):e70035. doi: 10.1111/jorc.70035 (PMC12576761; doi:10.1111/jorc.70035)
Supplement: Supplementary file 1 — Supporting Information. [file JORC-51-0-s001.docx]

| **SUPPLEMENTARY FILE 1 \| Clinical implication of the chain of cannulation and patient involvement** | | | | | |
| --- | --- | --- | --- | --- | --- |
| **Category** | **Subcategory** | **Reference** | **Clinical application using rope ladder** | **Clinical application using buttonhole** | **Patient involvement** |
| Planning cannulation | Maturation and cannulation in new AVF | Aitken et al. (2018) | May be used before fully developed AVF | Create tunnel tracks when AVF is mature (diameter >6mm) | Individual approach |
|  |  | Parisotto & Pancirova (2018) | No specific application using rope ladder | No specific application using buttonhole | Silence |
|  |  | Staaf et al. (2023) | No specific application using rope ladder | No specific application using buttonhole | Silence |
|  | Patient record | Aitken et al. (2018) | Cannulation plan is needed, use patient record to communicate | Communicate angle, depth, direction, use patient record to communicate | Describe patients individual choice. Design the needling plan to involve the patient in the their vascular access care |
|  |  | Parisotto & Pancirova (2018) | No specific application using rope ladder | No specific application using buttonhole | Silence |
|  |  | Staaf et al. (2023) | No specific application using rope ladder | Some units use photos of the cannulated vessel to show the direction of the needle and position of the arm | Silence |
|  | Education and experience | Aitken et al. (2018) | Lack of knowledge and confidence lead to area puncture | Expert cannulators are needed to perform buttonhole successfully. Hygiene education is crucial in buttonhole | Adapt tunnel track to patient preference if self-cannulation is performed |
|  |  | Parisotto & Pancirova (2018) | No specific application using rope ladder | Should be performed only by highly experienced staff | Silence |
|  |  | Staaf et al. (2023) | No specific application using rope ladder | To create tunnel tracks, experience is needed. | Silence |
|  | Patient information | Aitken et al. (2018) | No specific application using rope ladder | No specific application using buttonhole | Information helps to gain control and reduce anxiety. Contributes to successful cannulation |
|  |  | Parisotto & Pancirova (2018) | No specific application using rope ladder | No specific application using buttonhole | Silence |
|  |  | Staaf et al. (2023) | No specific application using rope ladder | No specific application using buttonhole | Patients are encouraged to call if feel pain or discomfort |
|  | Screening and control | Aitken et al. (2018) | No specific application using rope ladder | Infection risk should be assessed before buttonhole is used including screening for MRSA/MSSA and resistance. Decolonisation may also be effective. Root cause analysis should be performed in all bacteraemia of those using buttonhole. | No specific application |
|  |  | Parisotto & Pancirova (2018) | Silence | Silence | Silence |
|  |  | Staaf et al. (2023) | Silence | Silence | Silence |
| Pre-cannulation | Physical examination | Aitken et al. (2018) | No specific application using rope ladder | Look for hubbings, enlarged sites, infections, prolonged bleeding, skin integrity and pain/discomfort | Use the patient history |
|  |  | Parisotto & Pancirova (2018) | No specific application using rope ladder | No specific application using buttonhole | Silence |
|  |  | Staaf et al. (2023) | Use “see, listen and feel” to plan for the next cannulation site | No specific application using buttonhole | Silence |
|  | Hygiene routines | Aitken et al. (2018) | No specific application using rope ladder | Scab should be removed completely and not in hast using sterile blunt needle/tweezers. Do not soften scab before removal.  Use of antibacterial topical ointment is recommended all buttonhole users with increased risk of infection. These patients should also be screened for mupirocin resistant *S. aureus* | No specific application |
|  |  | Parisotto & Pancirova (2018) | No specific application using rope ladder | Disinfect before and after scab removal. Remove scab by moister it and wash it off with gauze | Silence |
|  |  | Staaf et al. (2023) | No specific application using rope ladder | Remove scab before cannulation both when blunt and sharp needles are used, one scab picker per site. Some units also use moisted gauze to remove scab. Disinfect before and after scab removal | Silence |
|  | Arm position | Aitken et al. (2018) | No specific application using rope ladder | Consistent arm position during track development and cannulation | Let patient recall the position of the arm and the sensation of needling |
|  |  | Parisotto & Pancirova (2018) | No specific application using rope ladder | No specific application using buttonhole | Silence |
|  |  | Staaf et al. (2023) | No specific application using rope ladder | Use the same arm position every time | Silence |
|  | Tourniquet | Aitken et al. (2018) | No specific application using rope ladder | Use of tourniquet should be consistent, all the time or not at all | No specific application |
|  |  | Parisotto & Pancirova (2018) | No specific application using rope ladder | No specific application using buttonhole | Silence |
|  |  | Staaf et al. (2023) | No specific application using rope ladder | No specific application using buttonhole | Silence |
|  | Choosing a cannulation site | Aitken et al. (2018) | Use the whole length of the vessel to avoid area puncture.  Place the new site 0,5-1 cm above the previous. Go back to the bottom when the top is reached. Use one or two sides of the vessel | 2-4 sites is possible. Sites should not be placed in curves, dips, on aneurysms and in abnormal skin integrity. If a new tunnel track cannot be used with blunt needles after 12 sessions, consider new sites | Where and how to insert the needle should occur in discussion with the patient |
|  |  | Parisotto & Pancirova (2018) | The same site should not be used within two weeks. Place the new site at least 3 mm from the previous. | More than two tunnel tracks can be created to change in between.  Creation of tunnel tracks takes 6-12 treatments. It is possible to create up to four sites. | Silence |
|  |  | Staaf et al. (2023) | Place the new site 0,5-2 cm from the previous | No specific application using buttonhole | Silence |
|  | Preventing pain | Aitken et al. (2018) | No specific application using rope ladder | May lead to less pain but evidence is lacking | Pain, from the patients perspective is more complex than a pain score |
|  |  | Parisotto & Pancirova (2018) | Silence | Silence | Silence |
|  |  | Staaf et al. (2023) | No specific application using rope ladder | No specific application using buttonhole | Patients apply analgesic themselves |
| During cannulation | How to needle | Aitken et al. (2018) | Needle insertion is decided during pre-needle assessment.  Rope ladder is a recommended technique | The needle should glide down the track without need of excessive force. The needler can hold the tubing instead of the wings.  Buttonhole is a recommended technique to those with a low infection risk.  Sharp needles should not be used after creation of the tunnel track | The choice of technique should be based on patients preferences and clinical conditions and an individual risk assessment. This also include those who self-cannulate. |
|  |  | Parisotto & Pancirova (2018) | Rope ladder recommended as first choice | Buttonhole does not allow for any margins therefore it is not recommended as first choice. | Silence |
|  |  | Staaf et al. (2023) | One unit recommend rope ladder as first choice | The majority of units recommend buttonhole to most patients  The skin should be held tight in some units but not in others. The needle may be inserted using wiggling and rotation while held by the tube  Sharp needles are used in some units to all patients every treatment and in others only when it is difficult inserting blunt needles | Silence |
|  | Type of needle | Aitken et al. (2018) | No specific application using rope ladder | Plastic cannulas may be used to develop tunnel tracks. Two units keep the cannula in place until the track is created. Evidence regarding this is limited so the method is not recommended | No specific application |
|  |  | Parisotto & Pancirova (2018) | No specific application using rope ladder | No specific application using buttonhole | Silence |
|  |  | Staaf et al. (2023) | No specific application using rope ladder | No specific application using buttonhole | Silence |
|  | Angle during cannulation | Aitken et al. (2018) | No specific application using rope ladder | No specific application using buttonhole | No specific application |
|  |  | Parisotto & Pancirova (2018) | No specific application using rope ladder | No specific application using buttonhole | Silence |
|  |  | Staaf et al. (2023) | No specific application using rope ladder | Use the same angle all the time, different angles may lead to false tracks | Silence |
|  | Fixating and adjusting | Aitken et al. (2018) | No specific application using rope ladder | Keep 1-2 mm of the needle visible to prevent from hubbings | No specific application |
|  |  | Parisotto & Pancirova (2018) | No specific application using rope ladder | No specific application using buttonhole | Silence |
|  |  | Staaf et al. (2023) | No specific application using rope ladder | Keep 2-3 mm of the needle visible to prevent from hubbings | Silence |
| Evaluating cannulation | Arterial and venous pressure | Aitken et al. (2018) | No specific application using rope ladder | No specific application using buttonhole | No specific application |
|  |  | Parisotto & Pancirova (2018) | No specific application using rope ladder | No specific application using buttonhole | Silence |
|  |  | Staaf et al. (2023) | No specific application using rope ladder | No specific application using buttonhole | Silence |
|  | Blood pump speed | Aitken et al. (2018) | No specific application using rope ladder | No specific application using buttonhole | No specific application |
|  |  | Parisotto & Pancirova (2018) | No specific application using rope ladder | No specific application using buttonhole | Silence |
|  |  | Staaf et al. (2023) | No specific application using rope ladder | No specific application using buttonhole | Silence |
| Post-cannulation | Needle withdrawal | Aitken et al. (2018) | Silence | Silence | Silence |
|  |  | Parisotto & Pancirova (2018) | No specific application using rope ladder | No specific application using buttonhole | Silence |
|  |  | Staaf et al. (2023) | No specific application using rope ladder | No specific application using buttonhole | Silence |
|  | Haemostasis | Aitken et al. (2018) | Silence | Silence | Silence |
|  |  | Parisotto & Pancirova (2018) | No specific application using rope ladder | No specific application using buttonhole | Silence |
|  |  | Staaf et al. (2023) | No specific application using rope ladder | No specific application using buttonhole | Silence |
| The new subcategory ‘screening and control’ is added as well as ‘patient involvement’ which can be included in all categories. Grey areas describe the different guidelines special recommendations regarding clinical approach to the steps in chain of cannulation describing buttonhole, rope ladder and how to involve the patient. | | | | | |
